# Characterization of sulfhydryl oxidase from *Aspergillus tubingensis*

**DOI:** 10.1186/s12858-017-0090-4

**Published:** 2017-12-08

**Authors:** Outi Nivala, Greta Faccio, Mikko Arvas, Perttu Permi, Johanna Buchert, Kristiina Kruus, Maija-Liisa Mattinen

**Affiliations:** 1 VTT Technical Research Centre of Finland, Ltd., P.O. Box 1000, FI-02044 Espoo, Finland; 2 Independent scientist, St. Gallen, CH Switzerland; 3 Institute of Biotechnology, University of Helsinki, P.O. Box 65, FI-00014 Helsinki, Finland; 4 Department of Biological and Environmental Sciences, Nanoscience Center, University of Jyväskylä, P.O. Box 35, FI-40014 Jyväskylä, Finland; 5 Department of Chemistry, Nanoscience Center, University of Jyväskylä, P.O. Box 35, FI-40014 Jyväskylä, Finland; 6 Natural resources institute Finland (Luke), P.O. Box 2, FI-00790 Helsinki, Finland; 7 Department of Forest Products Technology, Bioproduct Chemistry, Aalto University, School of Chemical Technology, P.O. Box 16300, FI-00076 Espoo, Finland

**Keywords:** Secreted sulfhydryl oxidase, Dithiol oxidase, Aspergillus tubingensis, Glutathione oxidation, Nonribosomal peptide synthesis, Secondary metabolism

## Abstract

**Background:**

Despite of the presence of sulfhydryl oxidases (SOXs) in the secretomes of industrially relevant organisms and their many potential applications, only few of these enzymes have been biochemically characterized. In addition, basic functions of most of the SOX enzymes reported so far are not fully understood. In particular, the physiological role of secreted fungal SOXs is unclear.

**Results:**

The recently identified SOX from *Aspergillus tubingensis* (AtSOX) was produced, purified and characterized in the present work. AtSOX had a pH optimum of 6.5, and showed a good pH stability retaining more than 80% of the initial activity in a pH range 4-8.5 within 20 h. More than 70% of the initial activity was retained after incubation at 50 °C for 20 h. AtSOX contains a non-covalently bound flavin cofactor. The enzyme oxidised a sulfhydryl group of glutathione to form a disulfide bond, as verified by nuclear magnetic resonance spectroscopy. AtSOX preferred glutathione as a substrate over cysteine and dithiothreitol. The activity of the enzyme was totally inhibited by 10 mM zinc sulphate. Peptide- and protein-bound sulfhydryl groups in bikunin, gliotoxin, holomycin, insulin B chain, and ribonuclease A, were not oxidised by the enzyme. Based on the analysis of 33 fungal genomes, SOX enzyme encoding genes were found close to nonribosomal peptide synthetases (NRPS) but not with polyketide synthases (PKS). In the phylogenetic tree, constructed from 25 SOX and thioredoxin reductase sequences from IPR000103 InterPro family, AtSOX was evolutionary closely related to other Aspergillus SOXs. Oxidoreductases involved in the maturation of nonribosomal peptides of fungal and bacterial origin, namely GliT, HlmI and DepH, were also evolutionary closely related to AtSOX whereas fungal thioreductases were more distant.

**Conclusions:**

AtSOX (55 kDa) is a fungal secreted flavin-dependent enzyme with good stability to both pH and temperature. A Michaelis-Menten behaviour was observed with reduced glutathione as a substrate. Based on the location of SOX enzyme encoding genes close to NRPSs, SOXs could be involved in the secondary metabolism and act as an accessory enzyme in the production of nonribosomal peptides.

**Electronic supplementary material:**

The online version of this article (10.1186/s12858-017-0090-4) contains supplementary material, which is available to authorized users.

## Background

Sulfhydryl oxidases (SOXs) are flavin-dependent enzymes that catalyse the oxidation of free thiol groups to disulfide bonds with the concomitant reduction of molecular oxygen to hydrogen peroxide. SOXs have been isolated from animal and microbial sources [1–4]. Both intracellular and secreted enzymes have been reported [5–9]. SOX enzymes can be classified to four major families based on their structural features: intracellular single-domain proteins essential for respiration and vegetative growth / augmenter of liver regeneration (the Erv/Alr family), the endoplasmic reticulum oxidase (Ero) family, the quiescin-sulfhydryl oxidase (QSOX) family with multi-domain enzymes, and the secreted fungal SOXs family with FAD-dependent dimeric single-domain enzymes (reviewed in [10]). The secreted fungal SOXs differ from the members in other SOX families. They carry features from thioredoxin reductase and pyridine nucleotide flavin disulphide oxidoreductase sequences [10]. Many fungal species such as *Aspergillus niger, Penicillium chrysogenum, Aspergillus oryzae*, *Aspergillus sojae,* and *Calodon sp.* have been reported to secrete SOXs (glutathione oxidase EC 1.8.3.3) [1, 3, 5, 6, 10, 11]. Numerous SOX-coding genes have been found in the genome of fungi. The genome of the yeast *Saccharomyces cerevisiae* contains two putative genes encoding SOX enzymes, while so far published genomes of the fungal genus *Aspergilli* typically contain from 10 to 12 putative genes encoding SOX enzymes [12]. The genome of *A. tubingensis* contains genes encoding eight putative SOXs [13].

SOXs are attractive catalysts for industrial applications. They have been tested in food applications such as in dairy and baking. SOXs have been used for ultra-high temperature (UHT)-treated milk to remove of unpleasant flavour [6, 14]. In baking, SOX enzymes have been utilized to strengthen the structure of dough and improving bread properties [15–17]. SOXs have been suggested to reduce the allergenicity of pharmaceuticals [18]. The characterized fungal SOXs are known to crosslink bonds between peptides but not between proteins [1, 3, 19]. There occurs variation in the substrate specificity within SOXs from different sources. The small thiol-containing molecules are typical substrates for secreted fungal SOXs, whereas mammalian bovine SOXs are known to oxidase protein-bound cysteine residues [1, 10, 20]. The function of intracellular *S. cerevisiae* SOXs and of some multi-domain SOXs is elucidated but the role for secreted fungal SOXs is still unclear [7, 21]. The sectered fungal SOXs do not form a mixed disulphide intermediate on the contrary to the mammalian SOXs and intracellular *S. cerevisiae* SOXs (Erv1p, Erv2p) [22–24], which implies that secreted fungal SOXs are not directly involved in the oxization of the reduced proteins. Multi-domain SOXs from the quiescin-sulfhydryl oxidase (QSOX) family have been proposed to be involved in the formation of the extracellular matrix, in the maturation of proteins along the secretory pathway, and to act as antimicrobial agent [4, 21, 25, 26]. Extracellular QSOXs are multi-domain sulfhydryl oxidases from multicellular organisms, and greatly differ from the single-domain secreted fungal SOXs. The QSOX and secreted fungal SOX proteins belong to different protein families and appear to be evolutionary unrelated. In bacteria, SOXs might be involved in the synthesis of bioactive compounds such as nonribosomal peptides [27].

This paper reports the biochemical characterization of SOX from *A. tubingensis* (AtSOX) previously identified in the screening study [28]. AtSOX was purified and its substrate specificity was further characterized and its activity on different peptide- and protein-bound sulfhydryl groups analysed. The formation of enzyme-catalysed disulphide bond in glutathione was confirmed by NMR spectroscopy. Furthermore, 33 fungal genomes were analysed to examine location of the selected genes, for example nonribosomal peptide synthetase or polyketide synthase coding genes, in relation to SOX-coding genes.

## Methods

### Production and purification of AtSOX

*Aspergillus tubingensis* strain D-85248 was obtained from the VTT Culture Collection [29] and cultivated as described in [28]. *A. tubingensis* was grown on PeptoneTM-D(+)-glucose media adapted from [3, 5] in liquid cultivation at 30 °C under shaking (250 rpm). After removal of the fungal biomass by filtration, AtSOX was purified from the cell-free extract with four chromatographic steps according to the procedure described below. The culture supernatant was concentrated and buffer exchanged to 20 mM Tris-HCl pH 7 with a PD10 column (no 17-0851-01, GE Healthcare, Uppsala, Sweden), and applied on a QSepharose fast flow column (volume = 20 mL, Amersham Biosciences, Piscataway, USA). Proteins were eluted with a linear gradient from 0 to 0.3 M NaCl in 20 mM Tris-HCl pH 7. The SOX activity of the fractions was detected using 5,5-dithio-bis(2-nitrobenzoic acid) (Ellman’s reagent, DTNB, no D 218200, Sigma-Aldrich, Helsinki, Finland) and oxygen consumption measurements. The SOX active fractions were pooled and applied to a Superdex 75 column (volume = 24 mL, Amersham Biosciences, USA) using 50 mM Tris-HCl pH 7 buffer containing 150 mM NaCl at a 0.1 mL/min flow rate. SOX-active fractions were pooled, concentrated, buffer exchanged to 20 mM Tris-HCl pH 7, and applied twice to a Resource Q Sepharose column (volume = 1 mL, Amersham Biosciences, USA). In the first separation using Resource Q Sepharose resin, the proteins were eluted with a linear gradient from 0 to 0.2 M NaCl in 20 mM Tris-HCl pH 7, whereas in the second separation a shallower gradient from 0 to 0.11 M NaCl was used. Prepacked anion exchange columns were connected to ÄKTA™ chromatography system and UNICORN Control Software (GE Healthcare, Uppsala, Sweden).

Protein concentration was determined using a Bio-Rad DC protein assay kit (Bio-Rad, Hercules, USA) and bovine serum albumin (BSA, no. A8022, Sigma, St. Louis, USA) as a standard. Proteins were analysed by ready-made 12% Tris-HCl SDS-PAGE gel (no 161-1156, Bio-Rad, Hercules, USA) to ensure purity of the isolated AtSOX enzyme.

### Amino acid analysis of AtSOX and strain identification

The partial amino acid sequence of AtSOX was determined from the N-terminus and internal peptide fragments. SDS-PAGE protein bands were stained with Coomassie Brilliant Blue, and the protein band of interest was excised and subjected to N-terminal sequencing. Edman degradation was performed using a Procise 494A protein sequencer from Perkin Elmer, Applied Biosystems Division (Foster City, CA, USA). The Coomassie stained protein band, assumably containing AtSOX, was cut off from the SDS-PAGE and in gel digested essentially as described by [30] for obtaining internal sequences and for peptide mass fingerprinting (PMF). In gel digestion was done by reducing proteins with dithiothreitol (DTT), alkylating with iodoacetamide and digesting with trypsin (no V5111, Promega, Madison, USA). The enzymatic cleavage occurred during overnight incubation at 37 °C. The peptides produced by enzymatic cleavage were analysed by MALDI-TOF MS after desalting using mC18 ZipTip (no ZTC18M096, Millipore, Billerica, USA). MALDI-TOF mass spectra of peptide fragments for PMF were obtained using an Ultraflex TOF/TOF instrument (Bruker-Daltonik GmbH, Bremen, Germany) using α-cyano-4-hydroxycinnamic acid (CHCA) as matrix. The sample solution was pipetted onto the sample plate together with matrix and air-dried. An electrospray ionization quadrupole time-of-flight tandem mass spectra for de novo sequencing were acquired using a Q-TOF instrument (Micromass, Manchester, UK) as described by [31]. Protein identification with the generated data, the obtained peptide masses and the partial sequences, was performed using the Mascot Peptide Mass Fingerprint and MS/MS Ion Search programmes (http://www.matrixscience.com). Identification of the production strain (VTT D-85248) based on the morphology and DNA sequencing was performed at the Identification Services CBS (Utrecht, The Netherlands).

### Peptide and protein substrate preparation

The reduced glutathione (GSH, no G4251), urinary trypsin inhibitor fragment (bikunin, no U-4751), and ribonuclease A (RNase A, no R5500) were purchased from Sigma (St. Louis, USA). Peptides, gliotoxin (no. A7665) and holomycin (no. sc-49,029), were purchased from PanReac AppliChem GmbH (Darmstadt, Germany) and Santa Cruz Biotechnology Inc. (Dallas, USA), respectively. The insulin B chain as Bunte salt derivative was kindly provided by Dr. Elisabeth Heine from Deutsches Wollforschungsinstitut (DWI, Aachen, Germany). Insulin chain B and RNaseA were reduced before use with 3% (v:v) 2-mercaptoethanol in the presence of 8 M urea according to the method used by [3]. For the reduction, insulin B was dissolved in 50 mM (NH_4_)HCO_3_ pH 8.3 containing 8 M urea and 3% (v:v) 2-mercaptoethanol was used as solvent. In the case of RNaseA, 200 mM Tris-HCl buffer (pH 7.4) with urea and 2-mercaptoethanol was used. The solutions were incubated overnight at room temperature. The final concentration of insulin chain B was 1 mM, and concentration of RNaseA was 0.5 mM. After reduction the free sulfhydryl groups were detected with 5,5-dithio-bis(2-nitrobenzoic acid). Gliotoxin and holomycin were reduced with two equivalence of Tris(2-carboxyethyl)phosphine hydrochloride (TCEP-HCl) under nitrogen flow for 1 h prior liquid chromatography mass spectrometry (LC-MS) analysis.

### Assay of AtSOX activity and pH and temperature behaviour

Different methods were used to measure AtSOX activity. First DTNB [32] was used for the detection of free sulfhydryl groups in the peptides after the enzymatic reaction. The spectroscopic measurements were done with a Cary 100 Bio UV-vis spectrophotometer (Varian Inc., Houten, the Netherlands). Second, the oxygen consumption measurement with Fibox 3 PreSens fiber-optic oxygen meter (Presens GmbH, Regensburg, Germany) was used to measure the changes in the concentration of dissolved oxygen during enzymatic reaction as described by [28]. GSH (5 mM) was used as a substrate when determining AtSOX activity. The substrate was dissolved in phosphate buffered saline (PBS) containing 68 mM NaCl and 75 mM KH_2_PO_4_ pH 7.4. Activity of AtSOX was measured by the oxygen consumption assay in the presence of different GSH concentrations (0.25-10 mM) to determine the kinetic parameters. The Michaelis-Menten constant (K_m_) and maximum velocity (V_max_) were determined with graphing software GraphPad Prism (GraphPad Software Inc., San Diego, USA) using nonlinear curve fitting to the Michaelis-Menten equation.

The thermal stability of AtSOX was determined at 30, 40, 50, 60 and 70 °C. The enzyme preparation was incubated in McIlvaine buffer pH 6.5 in a 0.2 mg/mL protein concentration for 1, 2, 15.5 and 20 h at 30 - 70 °C and also for 15 min at 70 °C, and the residual activity was measured by the oxygen consumption assay. pH stability was determined for AtSOX in a pH range between 2.3 and 10, and the residual enzyme activity was analysed by the oxygen consumption assay after 1 and 20 h incubation. pH optimum was determined by measuring AtSOX activity with the oxygen consumption assay using glutathione in McIlvaine citrate/phosphate buffer (pH 2.3-7.5), 50 mM Tris-HCl (pH 7-9) and 50 mM Glycine-NaOH (pH 8.5-10).

### Spectroscopy measurements

UV-vis absorption spectra were measured in 20 mM Tris-HCl pH 7.5 at 20 °C using a Cary 100/300 UV-vis spectrophotometer (Varian Inc., Houten, the Netherlands). In order to release a flavin cofactor, enzyme was thermally denaturated at 100 °C for 15 min followed by centrifugation (13,000 rpm, 10 min). Fluorescence was measured at 20 °C with a Cary Eclipse Fluorescence Spectrophotometer (Varian Inc., Houten, the Netherlands) using AtSOX solution in 20 mM Tris-HCl pH 7.5 in quartz cuvette with four optical faces. FAD fluorescence was recorded by exciting at 450 nm and monitoring emission between 450 and 600 nm.

### Inhibition analysis of AtSOX

The AtSOX activity was analysed in a buffer solution and in the presence of different inhibitors. The effect of possible inhibitors on the AtSOX activity was determined using 5 mM GSH as substrate. The tested potential inhibitors were DTT, ethylenediaminetetraacetic acid (EDTA), potassium iodide, magnesium sulphate, manganese sulphate, sodium sulphate, sodium chloride, zinc sulphate, and sodium dodecyl sulphate (SDS). The inhibitors were tested at 10 mM concentration in 200 mM Tris-HCl buffer pH 7.5, and zinc sulphate was tested also at 1 mM concentration. The residual SOX activity was measured by oxygen consumption measurements (Fibox 3 PreSens fiber-optic oxygen meter, PreSens GmbH, Regensburg, Germany). Inhibition by zinc sulphate was confirmed by homovanillic acid (HVA) and peroxidase coupled assay as described in [33], and the assay was done according to [34] using GSH (5 mM) as a substrate. Chemicals, HVA (Cat. no. H1252) and peroxidase type II (Cat. no. P8250), were purchased from Sigma-Aldrich (St. Louis, USA). Fluorescence from the production of a HVA dimer was measured in a black 96-well microtiter plate at 320 nm excitation and 420 nm emission wavelengths using a Varioskan spectral scanning multimode microplate reader (Thermo Electron co., Vantaa, Finland).

### Activity of AtSOX with reduced peptides

The ability of AtSOX to oxidise the peptides carrying free sulfhydryl groups was analysed by following the oxygen consumption. The activity of AtSOX (112 nkat) was analysed using reduced RNase A (0.25 mM solution in 200 mM Tris-HCl pH 7.4), and reduced GSH (5 mM solution in 75 mM KH_2_PO_4_ pH 7.4) as substrates in a total 1.86 mL reaction volume. The enzyme reactions were monitored by following the oxygen consumption of the co-substrate. The reactions were performed at room temperature and monitored for 5-20 min. For NMR spectroscopy, the enzymatically treated GSH was prepared by incubating the substrate with AtSOX for 20 min while the dissolved oxygen was totally consumed, as assessed by oxygen consumption measurements. Freshly prepared and 3 day old GSH solutions (5 mM) were used as a control samples to assess possible auto-oxidation. Reaction mixtures were analysed with ^1^H NMR as well as with ^13^C heteronuclear single quantum correlation (^13^C-HSQC) spectroscopy. The enzyme treated sample and the control sample were prepared in 75 mM potassium phosphate pH 7.4 in Shigemi NMR tubes. The NMR spectra were recorded on Varian INOVA 600 MHz NMR spectrometer at 293 K. One dimensional ^1^H spectra were recorded with water pre-saturation at 25 °C along with the gradient enhanced ^13^C-HSQC spectroscopy at 20 °C [35]. Homonuclear Total Correlation Spectroscopy (TOCSY) experiments [36] were recorded to confirm the assignment.

Besides oxygen consumption method and NMR spectroscopy, the selected peptides were analysed with a MALDI-TOF MS on an Autoflex II spectrometer (Bruker Daltonik GmbH, Bremen, Germany) using CHCA matrix for peptides and sinapic acid (SA) for proteins. The reduced substrates GSH and insulin B were dissolved in 50 mM (NH_4_)HCO_3_, bikunin in 75 mM KH_2_PO_4_, and RNase A in 200 mM Tris-HCl pH 7.4. Purified SOX was used in the experiments in a dosage of 5.6 nkat (4.1 μg protein) and in case of insulin B and RNase A also in 56 nkat (41 μg protein). The enzymatic reactions were performed at 40 °C, except for bikunin that was incubated at room temperature for 20 h. Matrix solutions were prepared by dissolving CHCA or SA in a 1:1 solution of 0.1% trifluoroacetic acid and 100% acetonitrile. The spots for MALDI plate were prepared by using 1:1 proportion of matrix and sample. Typically 1 μl matrix and 1 μl sample were used for MALDI spot, where matrix was spotted first and dried before adding sample.

Reduced gliotoxin and holomycin were used in 200 μM final concentration in 0.1 M phosphate buffer (pH 6.5) for spectroscopic measurements. Gliotoxin and holomycin were used in 440 and 680 μM final concentrations, respectively, for LC-MS analysis. UV-vis absorption spectra from 200 to 600 nm were recorded with Varioskan spectral scanning multimode microplate reader (Thermo Electron co., Vantaa, Finland). The UV-vis absorption spectra from 200 to 600 nm was recorded for 10 min before and after addition of AtSOX (100 μl reaction volume). Reduced peptides incubated (50 min at ambient temperature) with AtSOX or with denaturated AtSOX were analysed with a ultra performance LC (UPLC) combined with a photodiode array detector and SYNAPT G2-S High Definition Mass Spectrometry (Waters, Milford Massachusetts, USA). One microliter of the sample was injected to a LC pre-column. LC-MS system was using a C18 Acquity UPLC VanGuard pre-column (2.1 × 5 mm, 1.7 μm,) and a C18 Acquity UPLC column (2.1 × 100 mm, 1.7 μm). All solvents used were spectral grade. Eluents were 5 mM ammonium acetate 0.1% formic acid in H_2_O (A) and in methanol (B). Elution was started with 10% B for 1 min, followed by a linear gradient from 10 to 100% B for 10 min and finally at 100% B for 2 min, with 0.4 mL min^−1^ flow. In these conditions reduced gliotoxin eluted from the LC column after 5.23 min and gliotoxin standard after 6.02 min. Elution times for reduced holomycin and holomycin standard were 1.69 and 3.74 min, respectively.

### Analysis of SOX-coding genes in fungal genomes

The search was carried out as described in [37]. In brief, scaffolds of 33 fungal genomes from [38] were divided in windows of 16 genes that overlapped with two genes. InterPro protein annotations of the genes were then used to look for windows that contained a flavin adenine dinucleotide (FAD)-dependent pyridine nucleotide-disulfide oxidoreductase (PNDR) that recognizes also SOX enzymes (InterPro: IPR000103), nonribosomal peptide synthetases (NRPS) (InterPro: IPR000873) or polyketide synthases (PKS) (InterPro: IPR001227) and cytochrome P450 monooxygenases (P450, InterPro: IPR001128) and/or Zn2Cys6 transcription factors (Zn2, InterPro: IPR001138). Searches were carried out and the results visualised with R essentially as described by [38].

### Phylogenetic analysis

A phylogenetic analysis of the selected 25 proteins from the protein family pyridine nucleotide-disulphide oxidoreductase, class-II (InterPro: IPR000103) was carried out. The selected protein sequences were obtained from UniProtKB except AtSOX sequence was retrieved from the genome of *A. tubingensis* from Joint Genome Institute (JGI) genome portal [39]. The alignment of the sequences was done with MAFFT [40], and alignment was trimmed with trimAl [41]. The aligned sequences were from Ascomycetes species except two proteins were bacterial origin, namely, DepH from *Chromobacterium violaceum* (UniProtKB: A4ZPY8) and HlmI from *Streptomyces clavuligerus* (UniProtKB: E2PZ87). A phylogenetic tree was constructed with FastTree [42] and visualised by Geneious version 10.0 created by Biomatters.

## Results

### Purification and biochemical characterization of AtSOX from *Aspergillus tubingensis*

Purification of AtSOX was performed in four chromatographic steps from the culture cell-free medium. Fractions containing SOX activity eluted at a 110-260 mM NaCl concentration from a Q Sepharose anion exchange column. Pooled active fractions were applied to a Superdex 75 column for separation by SEC. Further purification of AtSOX was achieved with high resolution anion exchange chromatography. The SOX containing fractions eluted at a 60-175 mM and 100 mM NaCl concentration from two sequential purification steps where Resource Q columns were used. The fraction with the highest specific SOX activity was applied on a Resource Q column once again. AtSOX containing fractions started to elute at a 100 mM NaCl concentration. The purification process was monitored by SDS-PAGE (Additional file 1), and the final yield of protein was 3% of the initial activity.

**Fig. 1:**
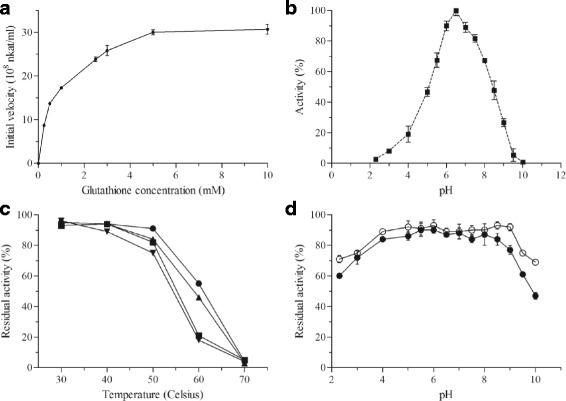
Kinetics, pH optimum and thermal and pH stability of AtSOX. AtSOX activity on GSH (0.25-10 mM) measured by the oxygen consumption assay (**a**). AtSOX activity was measured with the oxygen consumption assay on GSH (3 mM) at room temperature to determine the pH optimum (**b**) and temperature stability after 1 h (filled *circle*), 2 h (filled *triangle*), 15.5 h (filled *square*) and 20 h (filled inverted *triangle*) of incubation at different temperatures (30-70 °C) (**c**). pH stability of AtSOX as analysed by oxygen consumption measurements, when incubated within a pH range from 2.3 to 10 for 1 (empty *circles*) or 20 h (filled *circles*) (**d**). Each curve represents average of two replicates (standard deviation < 10%)

**Fig. 2:**
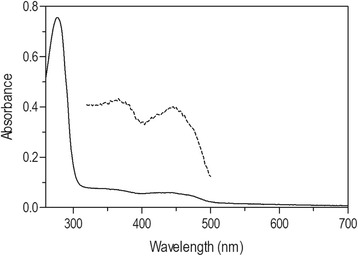
UV-visible spectrum of AtSOX enzyme. The absorbance spectra of AtSOX (continuous line) and of the released FAD (values 10×, *dashed line*) are shown

AtSOX preferred GSH (3 mM) as a substrate over cysteine and DTT. AtSOX had residual activity on L-cystein 14 ± 0.2%, on D-cystein 3 ± 1.2% and on DTT 9 ± 1.8% measured with oxygen consumption assay. Oxygen consumption in AtSOX-catalysed reaction using GSH (3 mM) as a substrate is shown in Additional file 2. Michaelis-Menten behaviour was observed for AtSOX using GSH as a substrate. Michaelis-Menten constant (K_m_) was 0.80 ± 0.09 mM and maximum velocity (V_max_) (33.15 ± 0.99) × 108 nkat/ml (Fig. 1a). AtSOX retained 89% of the initial activity after 20 h incubation at 40 °C (Fig. 1c). Under identical conditions at 50 and 60 °C, AtSOX showed 75% and 18% residual activity, respectively. At 70 °C, the enzyme was inactivated within 15 min. AtSOX was stable within a broad pH range as it retained 80-90% of the initial activity after 20 h incubation at pH range from 4 to 8.5 (Fig. 1d). The pH optimum of AtSOX was pH 6.5 (Fig. 1b). The enzyme activity was totally inhibited by 10 mM zinc sulphate in the assay conditions. The relative SOX activity was 4% with 1 mM zinc sulphate. The inhibition of zinc sulphate was confirmed with HVA-peroxidase coupled assay (Additional file 3). The other analysed compounds did not inhibit AtSOX activity, or had only minor effect on it, as residual activity was more than 90% in the presence of the analysed compound. For example the AtSOX retained 92% of its activity in 10 mM of potassium iodide solution and denaturant SDS did not inhibit enzyme activity. The flavoenzymatic nature of AtSOX was determined by spectral analyses using UV-vis and fluorescence spectrophotometry. The absorption spectra were recorded before and after thermal denaturation of AtSOX. The flavin cofactor was released by denaturation and detected in solution (Fig. 2). The UV-vis spectrum of AtSOX showed the characteristic peaks of flavoproteins with absorbance maxima at 275, 365 and 445 nm with a shoulder at 475 nm. AtSOX fluorescence emission spectrum had a peak at 525 nm when excited at 450 nm, which is characteristic for flavins.

**Fig. 3:**
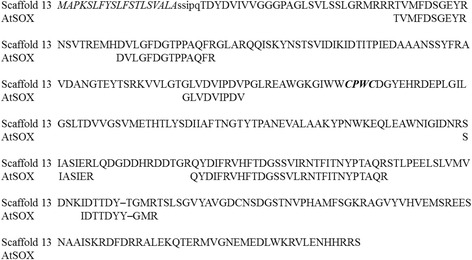
The reconstructed AtSOX sequence. Alignment of the peptides obtained by tryptic digestion of AtSOX and the protein they identify on scaffold 13 of the genome of *A. tubingensis* [13]. The predicted signal sequence is in italic, the experimentally determined N-terminus of AtSOX is in lower case and the catalytic di-cysteine motif is bold in italic

The purified AtSOX was subjected to Edman degradation, and trypsin digestion to perform peptide mass fingerprinting (PMF), and to identify the protein. The PMF gave six peptides made of more than six amino acids (Fig. 3). Mining the available genome of A. tubingensis [39] with the sequences of the identified peptides allowed the identification of a single SOX-coding gene on scaffold 13. This gene coded for a 41.8 kDa secreted protein with a predicted N-terminus, after removal of the signal peptide, SSIPQ. This protein contained four of the six peptides identified from AtSOX, and the other two peptides were present but carry either an amino acid insertion or deletion. Due to the high sequence similarity with secreted SOX from *A. niger* (UniProtKB: A2QUK3), the strain was re-identified at the Identification Services CBS (Utrecht, the Netherlands). The identification results confirmed that the used VTT strain D-85248 was *A. tubingensis*.

### Oxidation of peptides and proteins with AtSOX

The ability of AtSOX to oxidise peptide-bound cysteine was tested with reduced model peptides and protein namely GSH, bikunin, insulin B chain, gliotoxin, holomycin and RNase A as substrates. Reaction products were analysed by MALDI-TOF MS and NMR spectroscopy. The MALDI-TOF MS analysis of the reaction mixtures showed a dimer (613 Da) formation with GSH after overnight enzymatic treatment whereas only a peak correspond to the monomer (308 Da) was detected in the mass spectrum of the untreated substrate. The enzymatic disulphide bond formation between GSH peptides was observed also with the spectroscopic measurements at 250 nm (Additional file 4). No dimer formation was detected with bikunin (919 Da), insulin B chain (3496 Da) or RNaseA (13,700 Da) as substrates by MALDI-TOF analysis. Other analysed small peptides – gliotoxin (326 Da) and holomycin (214 Da) – were not enzymatically oxidised as observed in the LC-MS analysis. Holomycin was auto-oxidised as oxidation was observed also with the denaturated AtSOX enzyme after 50 min incubation.

**Fig. 4:**
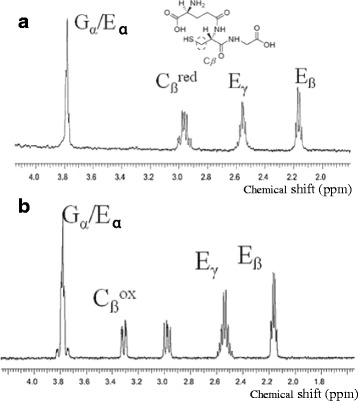
One dimensional ^1^H NMR spectra of reduced GSH (**a**) and GSH after incubation in the presence of AtSOX enzyme (**b**). The chemical structure of reduced GSH is shown as inset in (**a**)

**Fig. 5:**
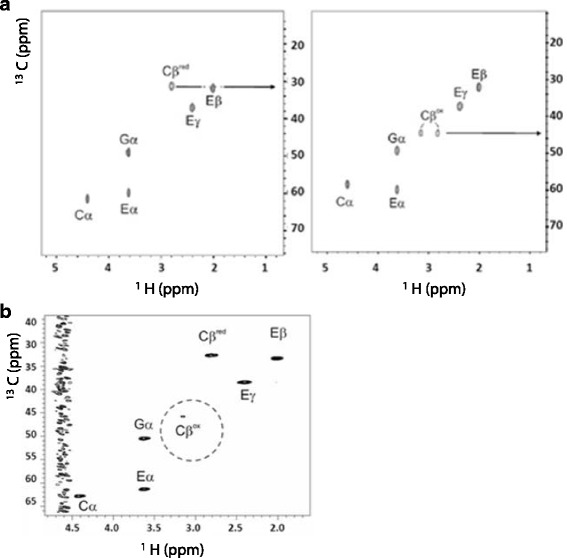
Oxidation of GSH by AtSOX as analysed by two-dimensional heteronuclear NMR spectroscopy (^13^C-HSQC spectra showing one-bond ^1^H-^13^C connectivities). The reduced substrate is shown on the left side as control and the enzyme-treated sample on the right side. The assignment of the tripeptide is indicated on the top of the cross-peaks. *Arrows* emphasize the chemical shift change in the C_β_ correlation of cysteine in the two conformations i.e. in the cross-linked and open form (**a**). The gradient selected ^13^C-HSQC spectrum of the substrate control to evaluate auto-oxidation three days after the preparation (**b**)

Oxidation of GSH and RNase A by AtSOX was also followed. AtSOX could oxidise only the tripeptide GSH, which was the shortest tested peptide. The final product formed in the reaction of AtSOX was analysed using NMR spectroscopy. Hereby, it was possible to follow the change in the cysteine oxidation state at atomic resolution. The oxidation was confirmed by following the oxygen consumption for 20 min after addition of AtSOX to the tripeptide L-GSH. The fresh GSH and enzyme-treated sample were analysed with one dimensional^1^H NMR. The one dimensional ^1^H spectra of GSH with and without AtSOX enzyme are shown in Fig. 4. Upon addition of AtSOX to GSH solution, the two degenerate protons bonded to C_β_ of cysteine residue became non-degenerated due to the formation of covalent disulphide bond. In addition, the two dimensional ^13^C-HSQC spectrum, exhibiting one-bond ^1^H-^13^C connectivities (Fig. 5a) showed a significant change in the ^13^C chemical shift of the Cys ^13^C _β_ -^1^H correlations as common for disulfide bond formation. This confirmed the fast dimerization of the GSH tripeptide through the disulfide bond upon addition of AtSOX. The control GSH substrate was checked once more 3 days after preparation with the gradient-enhanced ^13^C-HSQC spectrum to analyse auto-oxidation. The ^1^H, ^13^C cross peak, corresponding to the oxidised Cys C_ß_ form, started to appear which refers to dimerization (Fig. 5b). The dimerization of GSH occurred also without the SOX enzyme, although at a much slower rate. Measurement of the pure GSH sample 3 days after preparation showed an evidence of both monomeric and dimeric conformations in a ratio of about 5:1.

### SOX-coding genes in fungal genomes and phylogenetics

*Aspergilli* are known for their expanded secondary metabolism in comparison to yeasts [38], while fungi in general are known for their metabolic gene clusters, which are involved in, for example, synthesis of secondary metabolites [43] and catabolism of nutrients [37]. In order to suggest physiological roles for fungal secreted SOXs, fungal genomes were analysed. In brief, 33 fungal genomes were searched with custom R scripts for short genomic regions that would contain a SOX-coding genes and type of fungal secondary metabolism genes i.e. nonribosomal peptide synthetases (NRPS), polyketide synthases (PKS) and associated genes, i.e. cytochrome P450 monooxygenases (P450), and/or Zn2Cys6 transcription factors (Zn2).

**Fig. 6:**
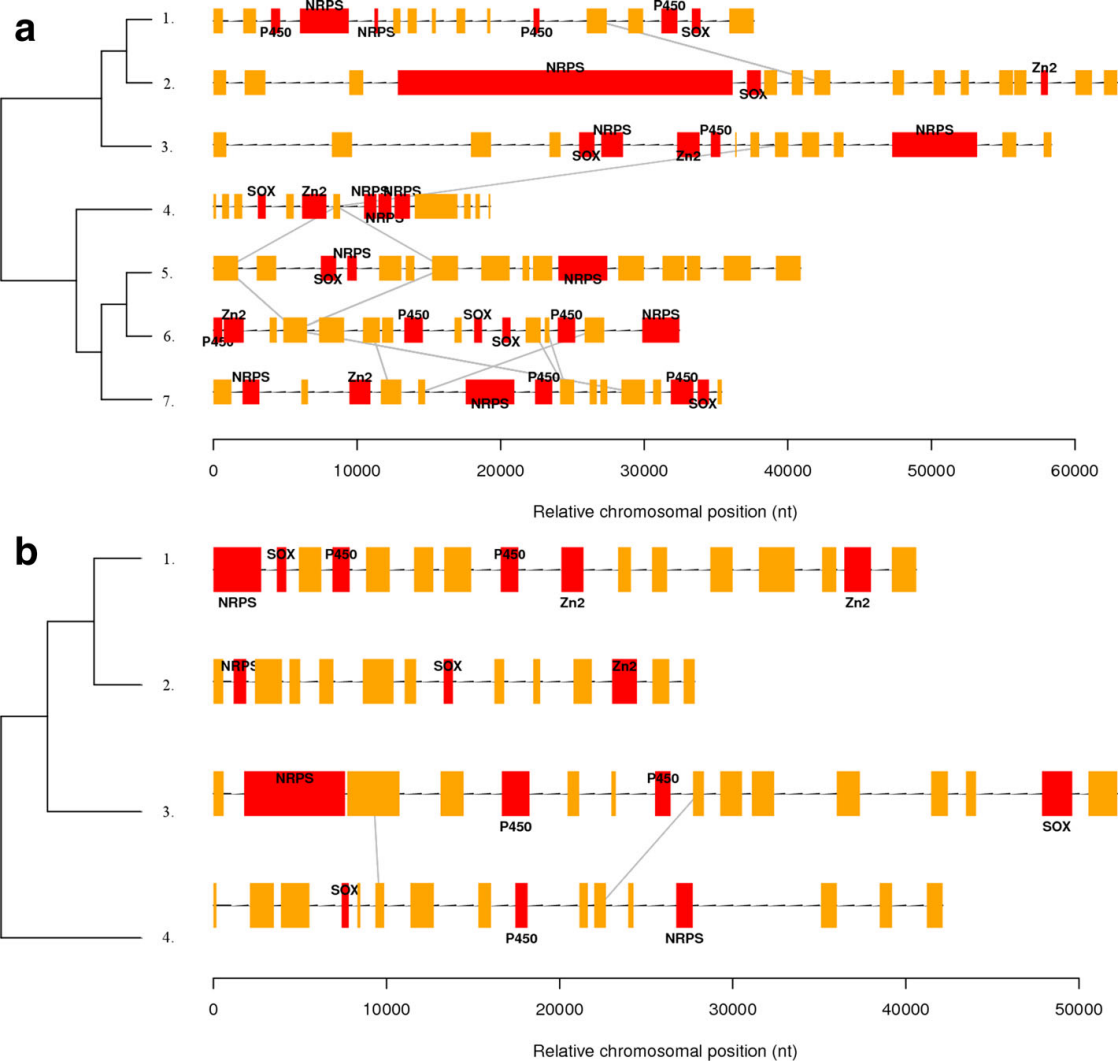
Candidate secondary metabolism clusters with SOX enzymes on fungal chromosomes. On the *left* an approximate phylogenetic tree of the species compiled from literature [36, 37]. On the *right* a stretch of a scaffold from each species containing the cluster and neighbouring genes. Genes are shown as *boxes* on the scaffold stretch. NRPS, PKS, P450 and Zn2 are indicated when present. *Grey lines* connect genes with identical protein domains on adjacent scaffolds (excluding NRPS, PKS, P450, Zn2 and SOX-coding genes) in order to reveal syntenies. Panel **a**. shows the gliotoxin clusters, while panel (**b**). shows other clusters. The strains in panel (**a**). are (1.) *Trichoderma reesei*, (2.) *Fusarium graminearum*, (3.) *Chaetomium globusum*, (4.) *Phaeosphaeria nodorum*, (5.) *A. tubingensis*, (6.) *A. oryzae* and (7.) *A. fumigatus*. The stains shown in panel (**b**). are (1.) *Fusarium graminearum*, (2.) *Magnaporthe grisea*, (3) *A. fumigatus* and (4.) *Phanerochaete chrysosporium*. Additional details are given in the Additional files 5 and 6

No genomic regions containing genes coding for PKS and SOX could be found, instead ten regions with SOX, NRPS and P450 or Zn2 coding genes were identified (Fig. 6). Two of these regions were the gliotoxin synthesis cluster of *A. fumigatus* [44] and the candidate gliotoxin cluster of *Trichoderma reesei* [45]. In addition, the *A. tubingensis* candidate gliotoxin cluster was detected (Fig. 6a). Based on protein clustering of SOX enzymes [38] the SOX enzymes found in the chromosomal gene clusters were divided in two groups: SOX enzymes of candidate gliotoxin gene clusters (Fig. 6a) and SOX enzyme of candidate secondary metabolism gene clusters (Fig. 6b).

**Fig. 7:**
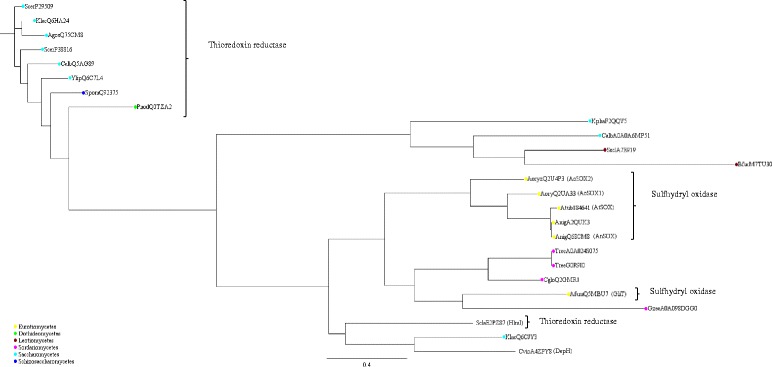
Phylogenetic tree including AtSOX. The phylogenetic tree of selected 25 SOX and thioredoxin reductase sequences from the IPR000103 protein family pyridine nucleotide-disulphide oxidoreductase, class-II (InterPro IPR000103). The sequences: AfumQ5MBU7, GliT SOX from *Aspergillus fumigatus* (UniProtKB: Q5MBU7); AgosQ75CM8, protein from *Ashbya gossypii* (UniProtKB: Q75CM8); AnigA2QUK3, SOX from *Aspergillus niger* (UniProtKB: A2QUK3); AnigQ68CM8, AnSOX from *Aspergillus niger* (UniProtKB: Q68CM8); AoryQ2UA33, AoSOX1 from *Aspergillus oryzae* (UniProtKB: Q2UA33); AoryQ2U4P3, AoSOX2 from *Aspergillus oryzae* (UniProtKB: Q2U4P3); Atub184641, AtSOX from *Aspergillus tubingensis* (JGI code 184641); BfucM7TU30, protein from *Botryotinia fuckeliana* (UniProtKB: M7TU30); CalbA0A0A6MP51, protein from *Candida albicans* (UniProtKB: A0A0A6MP51); CalbQ5AG89, protein from *Candida albicans* (UniProtKB: Q5AG89); CgloQ2GMR1, protein from *Chaetomium globosum* (UniProtKB: Q2GMR1); CvioA4ZPY8, DepH protein from *Chromobacterium violaceum* (UniProtKB: A4ZPY8); GzeaA0A098DGG0, protein from *Gibberella zeae* (UniProtKB: A0A098DGG0); KlacQ6CJY3, protein from *Kluyveromyces lactis* (UniProtKB: Q6CJY3); KlacQ6HA24, protein from *Kluyveromyces lactis* (UniProtKB: Q6HA24); KphaF2QQV5, protein from *Komagataella phaffii* (UniProtKB: F2QQV5); PnodQ0TZA2, protein from *Phaeosphaeria nodorum* (UniProtKB: Q0TZA2); ScerP29509, protein from *Saccharomyces cerevisiae* (UniProtKB: P29509); ScerP38816, protein from *Saccharomyces cerevisiae* (UniProtKB: P38816); SclaE2PZ87, HlmI protein from *Streptomyces clavuligerus* (UniProtKB: E2PZ87); SpomQ92375, protein from *Schizosaccharomyces pombe* (UniProtKB: Q92375); SsclA7E919, protein from *Sclerotinia sclerotiorum* (UniProtKB: A7E919); TreeA0A024S075, protein from *Trichoderma reesei* (UniProtKB: A0A024S075); TreeG0R9I0, protein from *Trichoderma reesei* (UniProtKB: G0R9I0); YlipQ6C7L4, protein from *Yarrowia lipolytica* (UniProtKB: Q6C7L4)

AtSOX sequence retrieved from *Aspergillus tubingensis* genome project was aligned with selected 24 SOX and thioredoxin reductase sequences from IPR000103 InterPro family, and the phylogenetic tree was constructed based on the alignment (Fig. 7). The C-X-X-C-motif was found from the majority of the aligned sequences (Additional file 7). It was observed from the phylogenetic tree that *Aspergillus* SOXs were evolutionary closely related. Oxidoreductases with activity on nonribosomal peptides, namely GliT, DepH and HlmI, were also closely related to AtSOX.

## Discussion

Various fungi and bacteria have been shown to secrete enzymes with a sulfhydryl oxidase activity. In this work, we focused on a secreted SOX from the fungus *A. tubingensis* and report the biochemical characterization of the secreted sulfhydryl oxidase AtSOX. *A. tubingensis* was previously identified as a natural SOX-producing strain [28]. In addition, we investigated the possible physiological role of SOX enzymes by bioinformatics means, as no clear role has yet been established for secreted fungal SOXs thus far. The SOX produced by *A. tubingensis* showed characteristics common to other secreted fungal SOXs. The small thiol group containing tripeptide GSH was a preferred substrate of AtSOX as earlier also reported for other SOXs of fungal origin [1, 3, 6, 19, 34]. The enzyme had a non-covalently bound FAD as a cofactor, which could be released by thermal denaturation of the protein, and the UV-vis spectrum of AtSOX showed the characteristic peaks of flavoproteins [3, 19, 34]. Inhibition studies with different salts showed that AtSOX activity was inhibited in the presence of zinc sulphate. Inhibition by zinc sulphate was observed also with secreted fungal SOXs from *A.oryzae* and *Penicillium* [3, 19, 34]. The catalytic center of SOX contains two reactive cysteine residues forming a C-X-X-C-motif [46, 47]. The cysteine residues are able to chelate divalent metal ions such as zinc, and the enzyme inhibition might be due to interact of zinc and cysteine as discussed in [23]. AtSOX showed good thermal stability up to 50 °C retaining 75% of its activity for 20 h. The thermostability of other known fungal SOX, AoSOX2 from *A. oryzae*, is on the same range [19].

The small thiol-containing molecules, like reduced GSH, cystein and DTT, are typical substrates for secreted fungal SOXs [3, 11, 19, 34]. The protein-bound thiols groups, particularly on reduced RNase A, have also been reported to be substrates for microbial SOXs from *A. niger* and *Penicillium* [1, 3]. Later analysis have shown that reported oxidation of RNaseA was spontaneous and non-enzymatic [23]. Our results also indicated that protein-bound cysteines in RNaseA (protein size 13.7 kDa) were not oxidised by AtSOX, but instead among the analysed compounds the tripeptide GSH was the preferred substrate. It was confirmed by NMR spectroscopy studies that the oxidation reaction of GSH was enzyme-catalysed. GSH was a good substrate for AtSOX based on the oxygen consumption analysis. AtSOX preferred GSH (K_m_ 0.8 mM) as a substrate over DTT, L-cysteine and D-cysteine. Other characterised SOXs from *A. oryzae* preferred cysteine as a substrate over GSH. AoSOX1 had smaller K_m_ for L-cystein (0.9 mM) than for GSH (2.78 mM), and AoSOX2 preferred D-cystein (K_m_ 1.55 mM) over GSH (K_m_ 3.7 mM) [19, 34]. The sulfhydryl groups in the longer peptides and protein-bound sulfhydryl groups were not oxidised suggesting preference for small substrates, which could have a better access into the active site of the enzyme. The QSOXs from an animal source, for instance bovine SOX, are known to oxidase protein-bound thiol groups and contribute in the oxidative protein folding [20], however secreted fungal SOXs are shown to have different substrate specificity and probably also a different physiological role. In this work, based on the genome analyses was suggested that secreted fungal SOXs might have role in the maturation of nonribosomal peptides.

It is not common for secondary metabolites of fungal and bacterial origin to contain disulphide bonds. Maturation mechanisms of these low-molecular mass metabolites are still unclear [48]. Thiol oxidising enzymes, oxidoreductases GliT, DepH and HlmI, have been reported to be involved in the maturation of gliotoxin from *A. fumigatus*, of the anticancer peptide romidepsin (FK228) in *Chromobacterium violaceum* No. 968, and of the antimicrobial compound holomycin in *Streptomyces clavuligerus*, respectively [48, 49]. A FAD-dependent SOX enzyme, GliT (dimeric ~ 60 kDa), belongs to the *A. fumigatus* gliotoxin chromosomal gene cluster, and it has been shown to form the atypical intramolecular disulfide bond responsible for toxicity of gliotoxin [44, 50]. GliT utilizes molecular oxygen as terminal electron acceptor with concomitant formation of hydrogen peroxide [51]. Similarly, a dimeric FAD-dependent pyridine nucleotide-disulfide oxidoreductase DepH (dimeric ~ 70 kDa; UniProtKB: A4ZPY8) has been found responsible for the introduction of a disulfide bond in the FDA-approved anticancer peptide FK228 from the Gram-negative *C. violaceum* [27]. Functionally homologous to GliT and DepH, a dimeric FAD-dependent thiol oxidising enzyme HlmI has been proven to be involved in the maturation of holomycin from *S. clavuligerus* [49]. DepH, GliT and HlmI shared common features with secreted fungal SOXs such as AoSOX1 and AoSOX2 (UniProtKB: Q2UA33, Q2U4P3), characterized by our group, and the reconstructed AtSOX sequence of this work (Fig. 3). They all carried sequence features of FAD-dependent pyridine nucleotide-disulfide oxidoreductase (InterPro: IPR013027 and the subclass II InterPro: IPR000103 used in this genome analysis study). AoSOX1 and AoSOX2 were proven FAD-dependent, dimeric and able to oxidise small thiol-containing peptide molecules such as GSH, cysteine and DTT. The reconstructed sequence of AtSOX shared 22-96% identity to other known secreted *Aspergillus* SOXs and oxidoreductases DepH, GliT and HlmI (Table 1). The flavoenzymes GliT, DepH and HlmI have a common reaction mechanism, although they have differences in substrate specificity [48]. The substrate binding clefts of GliT, DepH and HlmI enzymes are different but they all oxidise small molecules and introduce the disulfide bond to the corresponding secondary metabolites of fungi or bacteria [48].

**Table 1:** Biochemical characteristics of selected secreted flavin-dependent sulfhydryl oxidases and the enzymes DepH, GliT and HlmI reported to be involved in the secondary metabolism

	Biochemical properties						
Enzyme	MW (kDa)	pH optimum	pH stability	Temperature stability	Cofactor, non covalent	Identity (%) to AtSOX	Reference
AtSOX	55	6.5	> 80% activity after 20 h at pH 4-8.5	> 85% activity after 20 h at 40 °C	FAD	100	This study
AnSOX	53 (dimer)	5.5	n.a.	n.a.	FAD	96.0	[1]
AoSOX1	45 (dimer)	8.0	> 80% activity after 24 h at pH 5-8.5	> 70% activity after 24 h at 40 °C	FAD	64.6	[34]
AoSOX2	45 (dimer)	7.5-8.0	> 80% activity after 24 h at pH 5-8	> 65% activity after 1 h at 60 °C	FAD	46.5	[19]
DepH	34.4 (dimer)	7 (assay)	n.a.	n.a.	FAD	22.0	[27]
GliT	30 (dimer)	6.5 (assay)	n.a.	n.a.	FAD	24.5	[44, 51]
HlmI	39 (dimer)	6.5 (assay)	n.a.	n.a.	FAD	22.3	[49]

*Abbreviations*: *AtSOX* secreted SOX from *Aspergillus tubingensis*, *AnSOX* secreted SOX from *Aspergillus niger* (UniProtKB: Q68CM8), *AoSOX* secreted SOX from *Aspergillus oryzae* (AoSOX1 UniProtKB: Q2UA33, AoSOX2 UniProtKB: Q2U4P3), *DepH* enzyme from *Chromobacterium violaceum* (UniProtKB: A4ZPY8), *GliT* enzyme from *Aspergillus fumigatus* (UniProtKB: Q5MBU7), *HlmI* enzyme from *Streptomyces clavuligerus* (UniProtKB: E2PZ87), *MW* molecular weight of the subunit, *SOX* sulfhydryl oxidase, *n.a.* not available

Most of the secondary metabolites are derivatives from nonribosomal peptides and polyketides, the synthesis of which is catalysed by the multidomain enzymes belonging to NRPSs and PKSs [52]. Nonribosomal peptides are a class of molecules characterised by a vast structural and functional diversity, e.g. they can have linear, cyclic, or branched structures and activities ranging from antibiotic to metal-binding, from immunosuppressive to toxic and cytostatic [53]. Most of the fungal gene clusters for the secondary metabolite biosynthesis are silent under laboratory conditions [52]. The results (Fig. 6) indicated that SOX-coding genes are associated with NRPS, but not PKS, clusters and thus SOXs are possibly to act as accessory enzymes in the production of nonribosomal peptides. Thus, based on the genome studies, a connection between nonribosomal peptides and SOXs was suggested, since SOX and the nonribosomal peptide synthetase coding genes were found in the same clusters in the analysed fungal genomes. However, in vitro enzymatic oxidation of selected nonribosomal peptides (i.e. gliotoxin and holomycin) was not detected. This could be due to a challenging location of sulfhydryl groups in the circular structure of the peptides, and hence poor availability. Moreover flavoenzymes GliT, DepH and HlmI all have different substrate specificity. The cellular localisation, ability to oxidise small thiol-containing peptides, and genome comparison of secreted SOXs support the idea that fungal secreted SOXs are involved in the biosynthesis of disulfide-containing secondary metabolites, such as nonribosomal peptides. Their role could thus be in the maturation of peptides produced nonribosomally.

## Conclusions

This paper describes the characterization of a flavin-dependent secreted fungal SOX from *Aspergillus tubingensis* (AtSOX). AtSOX was shown to have good thermal stability and the enzyme retained high activity in the broad pH range. The enzyme preferred GSH as a substrate over the tested small-thiol containing molecules (reduced cysteine and DTT). The enzyme activity was drastically reduced in the presence of zinc sulphate. The enzymatic oxidation of the tripeptide GSH and formation of a disulphide bond was verified by nuclear magnetic resonance spectroscopy. AtSOX was evolutionary closely related to other Aspergillus SOXs and the oxidoreductases GliT, HlmI and DepH of fungal and bacterial origin, whereas fungal thioreductases were evolutionary more distant. Based on the location near to NRPSs encoding genes, SOXs could be involved in the secondary metabolism and act as an accessory enzyme in the production of nonribosomal peptides.

## Additional files

Additional file 1: Purification of AtSOX as analysed by SDS-PAGE. Molecular weight (MW) standards are shown in lanes 1 and 9. The sample from initial crude cell-free medium is shown in lane 2. As a first purification step was used anion exchange chromatography with a Q Sepharose column. In lane 3 are the unbound proteins, and in the lanes 4–6 bound and then eluted proteins, from Q Sepharose column. Lane 6: AtSOX containing fractions selected for further purifications steps. In the lanes marked 7 and 8 are shown fractions obtained from the last purification step using anion exchange chromatography with Resource Q column (analysed in a separate SDS-PAGE gel with MW standards in lane 9). (PPTX 137 kb)

Additional file 2: AtSOX activity measured by oxygen consumption assay using 3 mM reduced GSH as a substrate (continuous line). The reaction occurred at the enzymatic rate (the linear area ca. 0.5 - 3.5 min). The amount of dissolved oxygen in the reduced GSH solution prior addition of enzyme is shown with a dashed line. The triplicate measurements were done. (PPTX 6691 kb)

Additional file 3: AtSOX activity measured by HVA-peroxidase coupled assay using reduced GSH (5 mM) as a substrate according to [33]. The enzyme reaction is at the enzymatic rate (linear area ca. 0 - 150 s). The production of the fluorescent HVA dimer was followed at excitation wavelength 320 nm and emission wavelength 420 nm. The reduced AtSOX activity with the inhibitor zinc sulphate (10 mM) is also shown (dashed line). The triplicate measurements were done. (PPTX 121 kb)

Additional file 4: Absorbance spectra (ca. 10 min) of 5 mM reduced GSH (a.) and 5 mM reduced GSH with AtSOX (b.). Arrow indicates the direction of increased UV adsorption due to enzymatic oxidation of the substrate. (PPTX 395 kb)

Additional file 5: Details to Fig. 6 Candidate secondary metabolism clusters with SOX enzymes on fungal chromosomes. On the left an approximate phylogenetic tree of the species compiled from literature [54, 55]. On the right a stretch of a scaffold from each species containing the cluster and neighbouring genes. Genes are shown as boxes on the scaffold stretch. NRPS, PKS, P450 and Zn2 are indicated when present. Grey lines connect genes with identical protein domains on adjacent scaffolds (excluding NRPS, PKS, P450, Zn2 and SOX genes) in order to reveal syntenies. Codes above the gene boxes are their identifiers and below them the Interpro protein domain identifiers found in the genes. Panel a. shows the gliotoxin clusters, while panel b. shows other clusters. The strains shown in panel a. are *Trichoderma reesei*, *Fusarium graminearum*, *Chaetomium globusum*, *Phaeosphaeria nodorum*, *A. tubingensis*, *A. oryzae* and *A. fumigatus.* The stains shown in panel b. are *F. graminearum*, *Magnaporthe grisea*, *A. fumigatus* and *Phanerochaete chrysosporium*. (PNG 335 kb)

Additional file 6: For each chromosomal cluster the table shows accession numbers for genes (Accession), scaffold identifier (Scaffold), start and end on the scaffold, direction of the gene (Direction) and Interpro protein domain identifiers found in the genes (Interpro), as details for Additional file 5. (XLSX 20 kb)

Additional file 7: Part of the alignment of 25 sequences from the same protein family (InterPro IPR000103). On the first line is shown AtSOX retrieved from *A. tubingensis* genome. The C-X-X-C motifs are marked with a box. The sequences: AtSOX, secreted SOX from *A. tubingensis;* AnSOX, secreted SOX from *A. niger;* AoSOX, secreted SOX from *A. oryzae*; DepH, enzyme from *C. violaceum*; GliT, enzyme from *A. fumigatus*; HlmI, enzyme from *S. clavuligerus.* The other abbreviations are shown in the legend of Fig. 7. (PPTX 95 kb)

## Abbreviations

^13^C-HSQC: ^13^C heteronuclear single quantum correlation (spectroscopy)
AtSOX: Secreted SOX enzyme from *Aspergillus tubingensis*
CHCA: α-cyano-4-hydroxycinnamic acid
DTNB: 5,5-dithio-bis(2-nitrobenzoic acid), Ellman’s reagent
DTT: Dithiothreitol
EDTA: Ethylenediaminetetraacetic acid
FAD: Flavin adenine dinucleotide
GSH: The reduced glutathione
HVA: Homovanillic acid
MALDI-TOF MS: Matrix-assisted laser desorption-ionization time of flight mass spectrometry
NMR: Nuclear magnetic resonance (spectroscopy)
NRPS: Nonribosomal peptide synthetases
PKS: Polyketide synthases
PMF: Peptide mass fingerprinting
PNDR: Pyridine nucleotide-disulfide oxidoreductase
QSOX: The quiescin-sulfhydryl oxidase
Q-TOF: Quadruple time-of-flight
RNase A: Ribonuclease A
SDS: Sodium dodecyl sulphate
SEC: Size-exclusion chromatography
SOX: Sulfhydryl oxidase
TOCSY: Homonuclear total correlation spectroscopy
UPLC: Ultra performance liquid chromatography
