# Supplementary figures and images for: Characterization of sulfhydryl oxidase from Aspergillus tubingensis
Source: BMC Biochem. 2017 Dec 8;18:15. doi: 10.1186/s12858-017-0090-4 (PMC5721475; doi:10.1186/s12858-017-0090-4)

## Slide 1
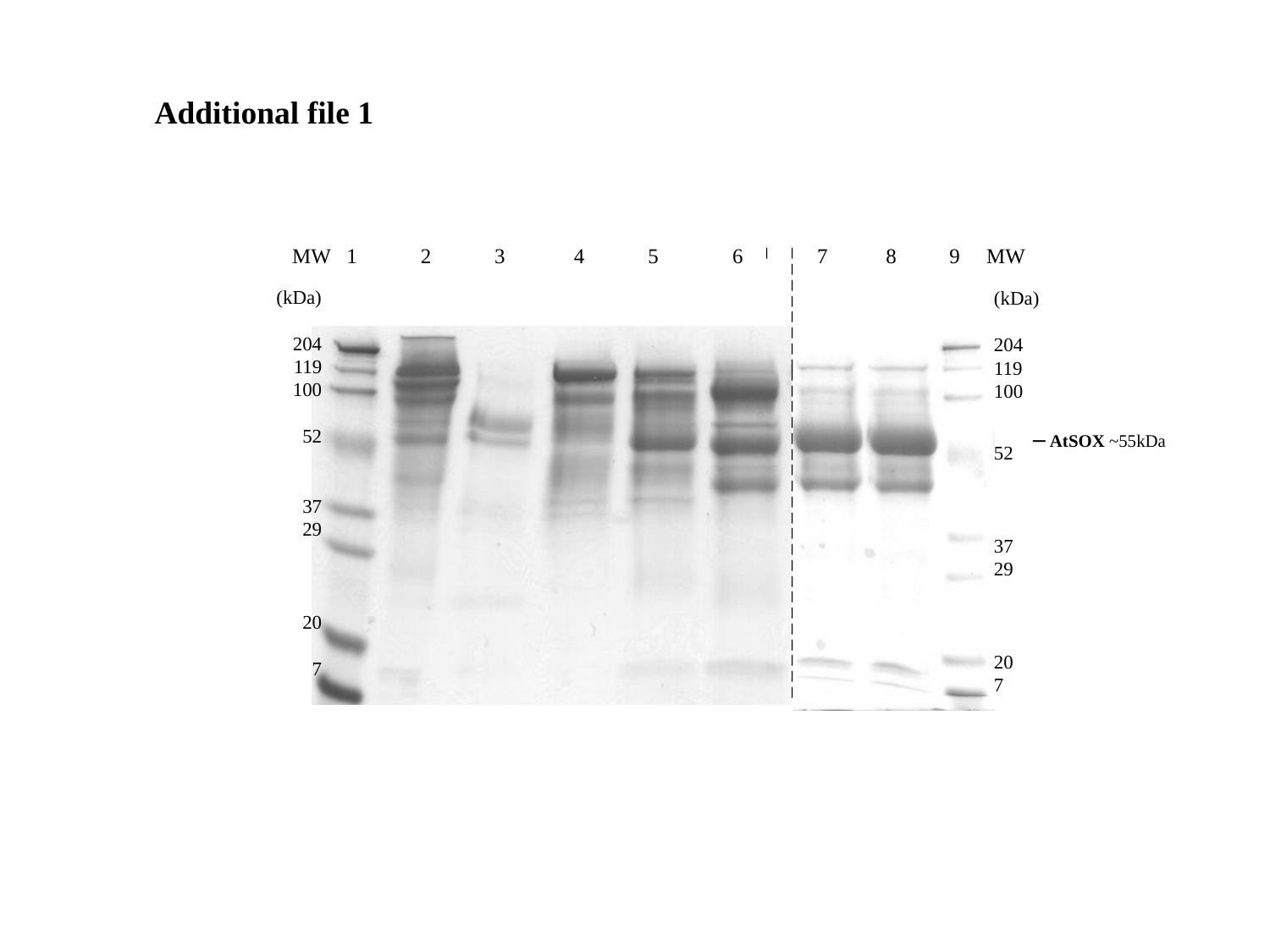

Additional file 1
MW 1 2 3 4 5 6 7 8 9 MW
(kDa)
204
119
100
52
37
29
20
7
– AtSOX ~55kDa
– – – – – – – –– – – – – – – – – – – – – – – – – – – – – –
(kDa)
204
119
100
52
37
29
20
7

Supplement: Supplementary file 1 — Purification of AtSOX as analysed by SDS-PAGE. Molecular weight (MW) standards are shown in lanes 1 and 9. The sample from initial crude cell-free medium is shown in lane 2. As a first purification step was used anion exchange chromatography with a Q Sepharose column. In lane 3 are the unbound proteins, and in the lanes 4–6 bound and then eluted proteins, from Q Sepharose column. Lane 6: AtSOX containing fractions selected for further purifications steps. In the lanes marked 7 and 8 are shown fractions obtained from the last purification step using anion exchange chromatography with Resource Q column (analysed in a separate SDS-PAGE gel with MW standards in lane 9). (PPTX 137 kb) [file 12858_2017_90_MOESM1_ESM.pptx]

## Slide 1
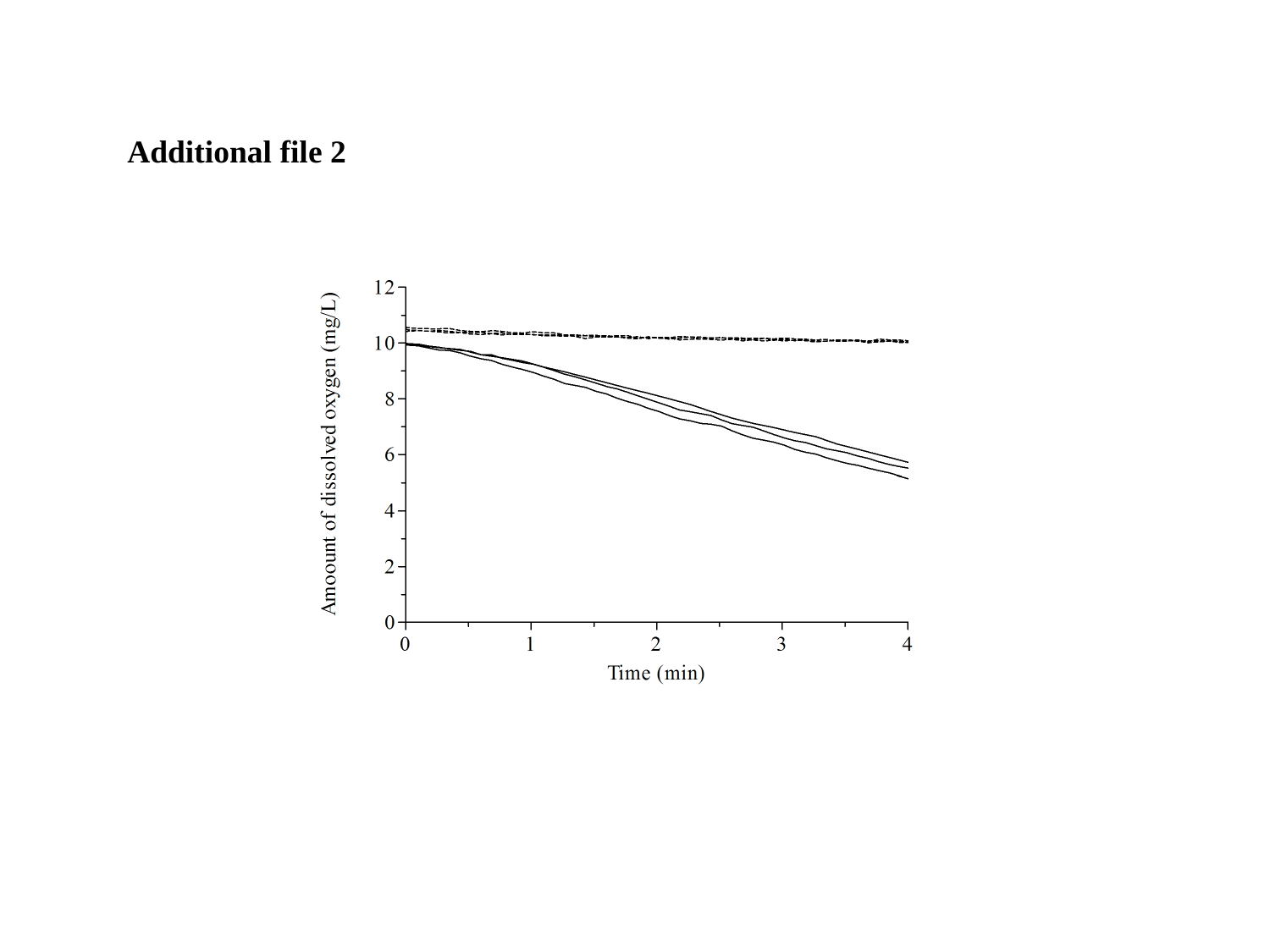

Additional file 2

Supplement: Supplementary file 2 — AtSOX activity measured by oxygen consumption assay using 3 mM reduced GSH as a substrate (continuous line). The reaction occurred at the enzymatic rate (the linear area ca. 0.5 - 3.5 min). The amount of dissolved oxygen in the reduced GSH solution prior addition of enzyme is shown with a dashed line. The triplicate measurements were done. (PPTX 6691 kb) [file 12858_2017_90_MOESM2_ESM.pptx]

## Slide 1
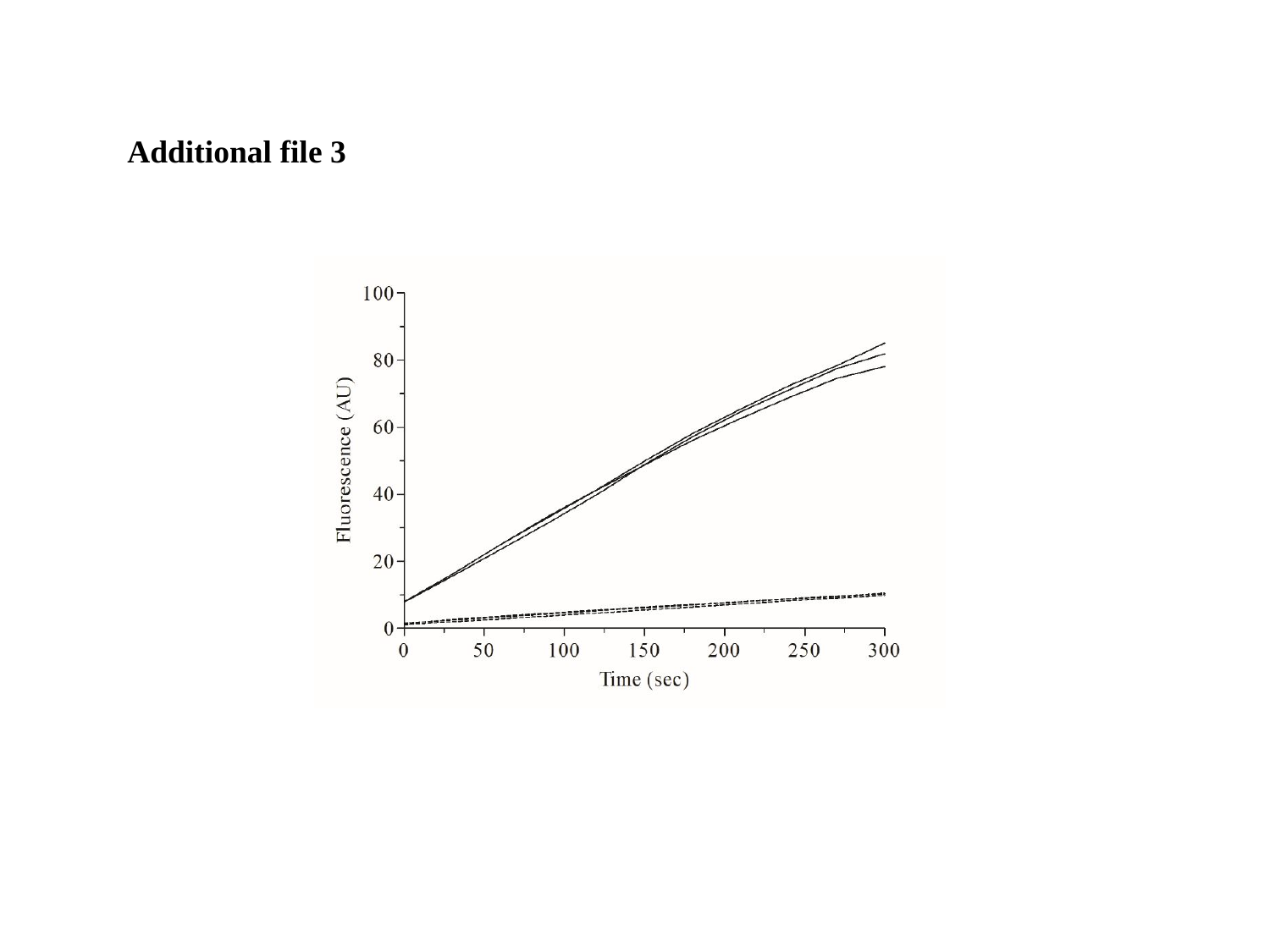

Additional file 3

Supplement: Supplementary file 3 — AtSOX activity measured by HVA-peroxidase coupled assay using reduced GSH (5 mM) as a substrate according to [33]. The enzyme reaction is at the enzymatic rate (linear area ca. 0 - 150 s). The production of the fluorescent HVA dimer was followed at excitation wavelength 320 nm and emission wavelength 420 nm. The reduced AtSOX activity with the inhibitor zinc sulphate (10 mM) is also shown (dashed line). The triplicate measurements were done. (PPTX 121 kb) [file 12858_2017_90_MOESM3_ESM.pptx]

## Slide 1
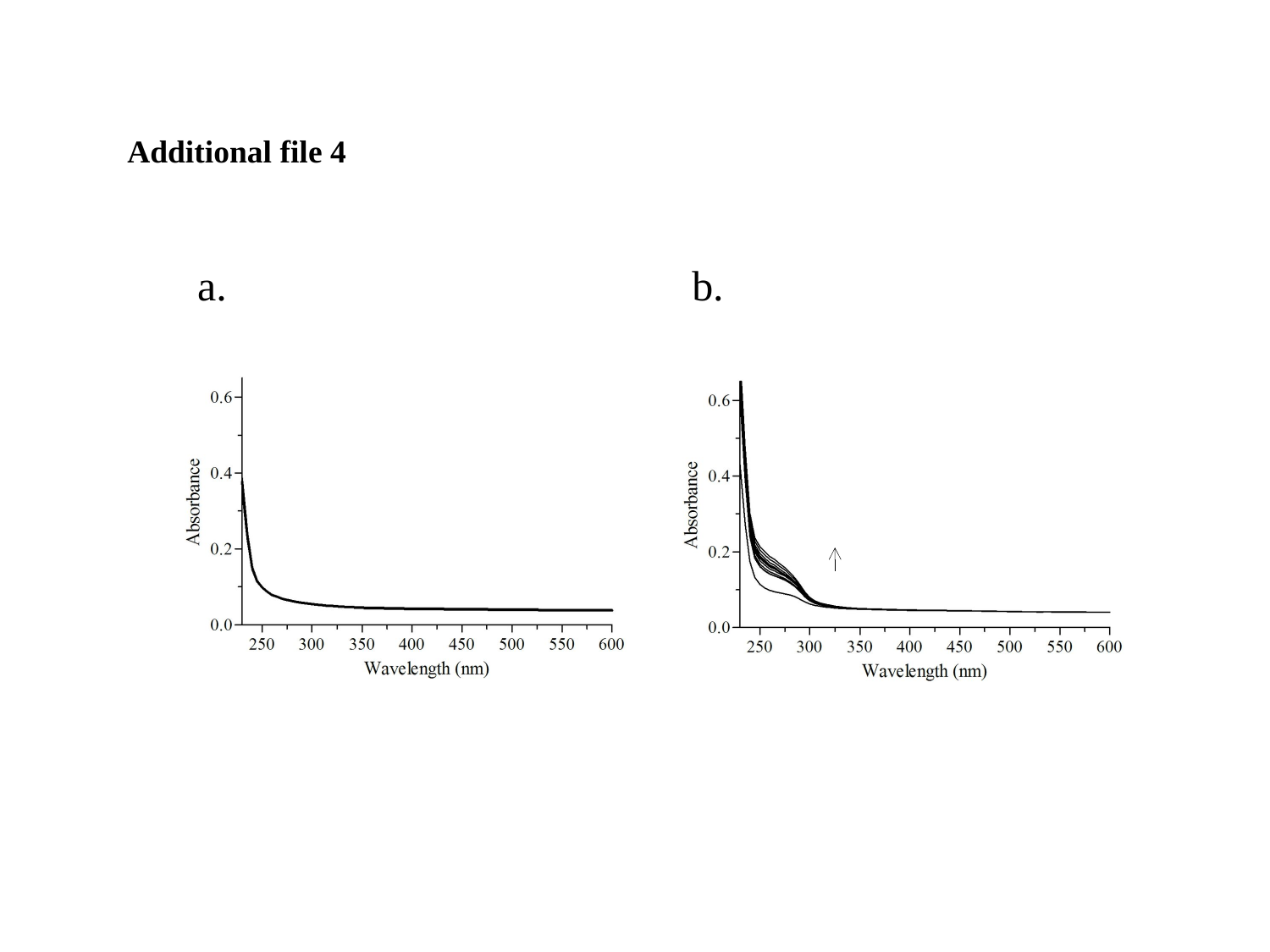

Additional file 4
a. b.

Supplement: Supplementary file 4 — Absorbance spectra (ca. 10 min) of 5 mM reduced GSH (a.) and 5 mM reduced GSH with AtSOX (b.). Arrow indicates the direction of increased UV adsorption due to enzymatic oxidation of the substrate. (PPTX 395 kb) [file 12858_2017_90_MOESM4_ESM.pptx]

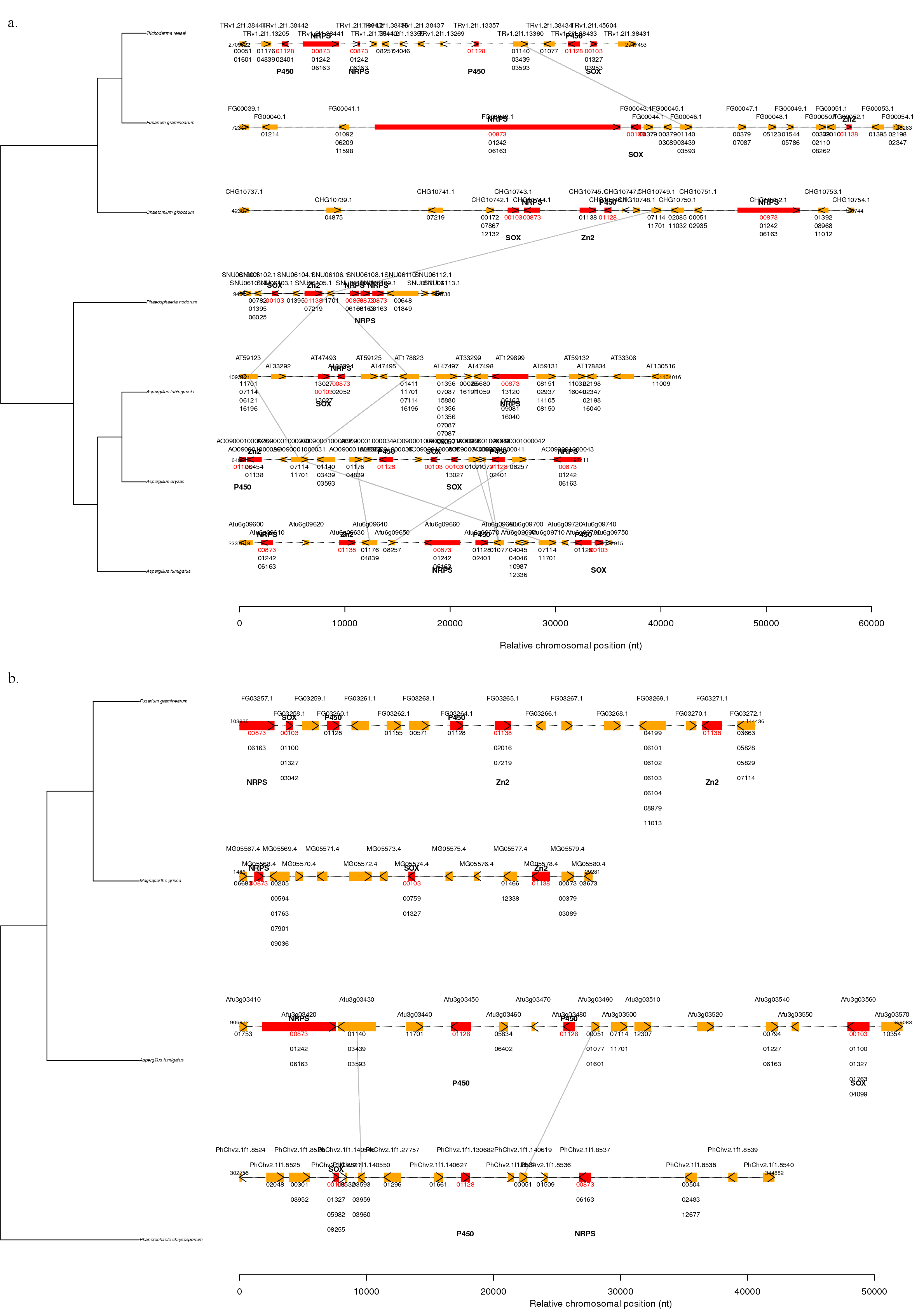

Supplement: Supplementary file 5 — Details to Fig. 6 Candidate secondary metabolism clusters with SOX enzymes on fungal chromosomes. On the left an approximate phylogenetic tree of the species compiled from literature [54, 55]. On the right a stretch of a scaffold from each species containing the cluster and neighbouring genes. Genes are shown as boxes on the scaffold stretch. NRPS, PKS, P450 and Zn2 are indicated when present. Grey lines connect genes with identical protein domains on adjacent scaffolds (excluding NRPS, PKS, P450, Zn2 and SOX genes) in order to reveal syntenies. Codes above the gene boxes are their identifiers and below them the Interpro protein domain identifiers found in the genes. Panel a. shows the gliotoxin clusters, while panel b. shows other clusters. The strains shown in panel a. are Trichoderma reesei, Fusarium graminearum, Chaetomium globusum, Phaeosphaeria nodorum, A. tubingensis, A. oryzae and A. fumigatus. The stains shown in panel b. are F. graminearum, Magnaporthe grisea, A. fumigatus and Phanerochaete chrysosporium. (PNG 335 kb) [file 12858_2017_90_MOESM5_ESM.png]

## Slide 1
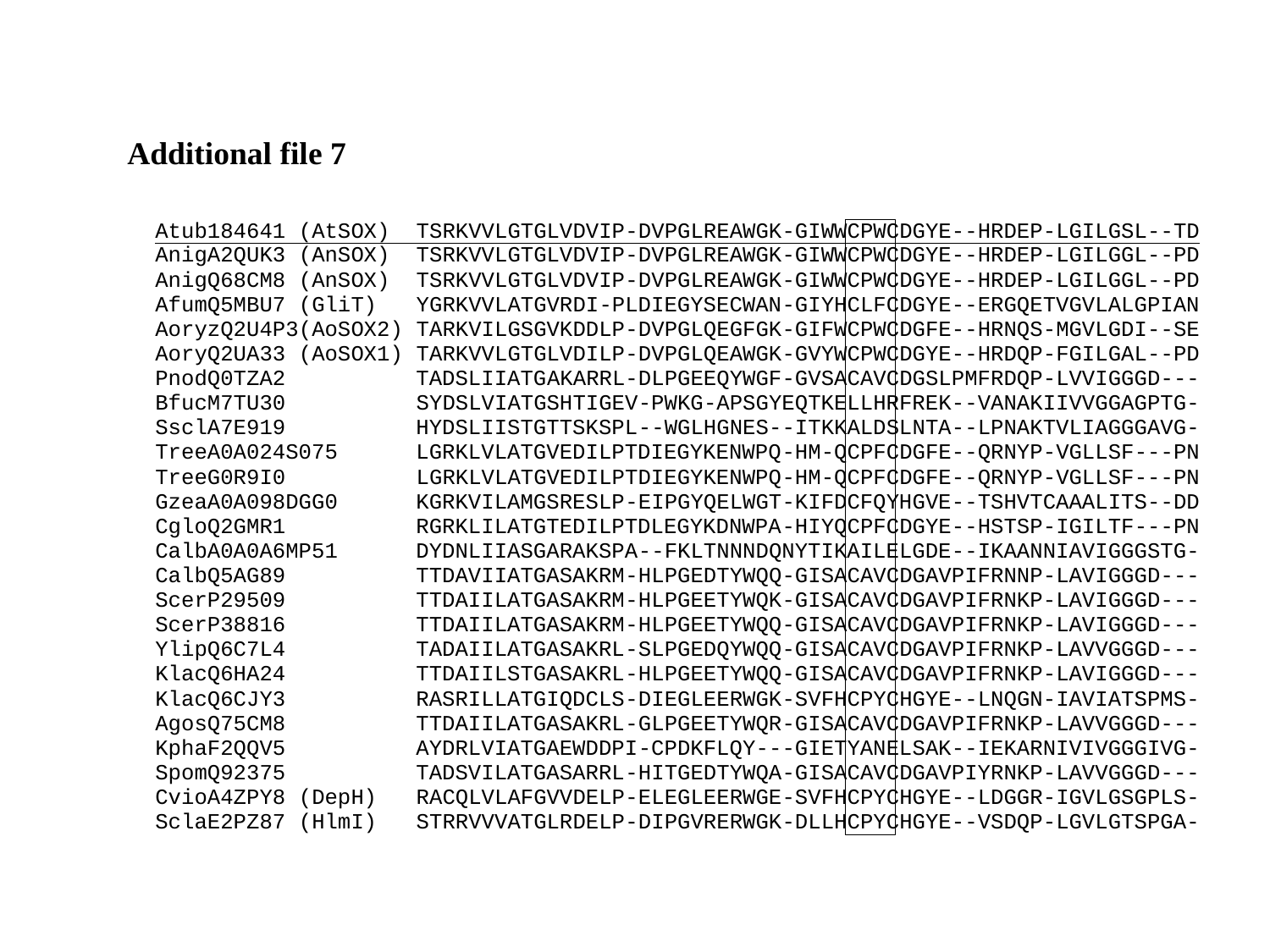

Additional file 7

Supplement: Supplementary file 7 — Part of the alignment of 25 sequences from the same protein family (InterPro IPR000103). On the first line is shown AtSOX retrieved from A. tubingensis genome. The C-X-X-C motifs are marked with a box. The sequences: AtSOX, secreted SOX from A. tubingensis; AnSOX, secreted SOX from A. niger; AoSOX, secreted SOX from A. oryzae; DepH, enzyme from C. violaceum; GliT, enzyme from A. fumigatus; HlmI, enzyme from S. clavuligerus. The other abbreviations are shown in the legend of Fig. 7. (PPTX 95 kb) [file 12858_2017_90_MOESM7_ESM.pptx]
